# The Validation of the ATRIA and CHA2DS2-Vasc Scores in Predicting Atrial Fibrillation after Coronary Artery Bypass Surgery

**DOI:** 10.21470/1678-9741-2019-0274

**Published:** 2020

**Authors:** Dinçer Uysal, Fatih Aksoy, Erdogan Ibrişim

**Affiliations:** 1 Department of Cardiovascular Surgery, Medical School, Suleyman Demirel University, Isparta, Turkey.; 2 Department of Cardiology, Medical School, Suleyman Demirel University, Isparta, Turkey.

**Keywords:** Coronary Artery Bypass, Atrial Fibrilation, Heart Atria, Postoperative Period, Anticoagulants, Drainage, Logistic Models

## Abstract

**Objective:**

The aim of this study was to evaluate the value of CHA_2_DS_2_-VASc and Anticoagulation and Risk Factors in Atrial Fibrillation (ATRIA) risk scores for prediction of postoperative atrial fibrillation (AF) development in patients undergoing coronary artery bypass grafting (CABG) operation.

**Methods:**

The population of this observational study consisted of 370 patients undergoing CABG operation. CHA_2_DS_2_-VASc and ATRIA risk scores were calculated for all patients and their association with postoperative AF (AF episode lasting > 5 min) were evaluated. Predictors of postoperative AF were determined by multiple logistic regression analysis.

**Results:**

During follow-up, 110 patients (29.7%) developed postoperative AF. With multiple logistic regression analysis, risk factors for postoperative AF were determined: ATRIA risk score (odds ratio [OR] 1.23; 95% confidence interval [CI] 1.11-1.36; *P*<0.001), fasting glucose level (OR 1.006; 95% CI 1.004-1.009; *P*<0.001), and 24-hour drainage amount (OR 1.002; 95% CI; 1.001-1.004; *P*<0.001). Receiver operating characteristic curve analyses showed that CHA_2_DS_2_-VASc and ATRIA risk scores were signiﬁcant predictors for new-onset AF (C-statistic 0.648; 95% CI 0.59-0.69; *P*<0.001; and C-statistic 0.664; 95% CI 0.61-0.71; P<0.001, respectively).

**Conclusion:**

CHA_2_DS_2_-VASc and ATRIA risk scores predict new AF in patients undergoing CABG.

**Table:** 

Abbreviations, acronyms & symbols				
**AF** **ALT** **AST** **ATRIA** **AUC** **CABG** **CI** **CVA** **HDL**	**= Atrial fibrillation** **= Alanine aminotransferase** **= Aspartate aminotransferase** **= Anticoagulation and Risk Factors in Atrial Fibrillation** **= Area under the curve** **= Coronary artery bypass grafting** **= Confidence interval** **= Cerebrovascular accident** **= High-density lipoprotein**		**IVSD** **LDL** **LVEDD** **LVESD** **OR** **POAF** **PWD** **ROC** **TIA**	**= Interventricular septum diameter** **= Low-density lipoprotein** **= Left ventricular end-diastolic diameter** **= Left ventricular end-systolic diameter** **= Odds ratio** **= Postoperative atrial fibrillation** **= Posterior wall diameter** **= Receiver operating characteristics** **= Transient ischemic attack**

## INTRODUCTION

Postoperative atrial fibrillation (POAF) is the most common type of arrhythmia after cardiac surgery with the incidence ranging from 10 to 65%, and its prevalence is even higher in elderly patients with heart failure and severe left ventricular impairment^[1]^. Patients with developing atrial fibrillation (AF) after cardiac surgery have higher risk for morbidity, such as cerebrovascular accidents, pulmonary edema, longer hospital stays, and mortality compared to those who do not develop AF^[1]^. Preoperative determination of patients who may develop POAF and taking necessary precautions will decrease the mortality and morbidity rates. Therefore, scoring systems should be developed to predict the development of POAF.

The CHA_2_DS_2_-VASc and Anticoagulation and Risk Factors in Atrial Fibrillation (ATRIA) risk scores are cheap and easy scoring systems that are used to predict the risk of thromboembolism in non-valvular AF patients^[2,3]^. Additionally, these scoring systems have been shown to accurately predict worse clinical outcomes in patients undergoing coronary artery bypass grafting (CABG) regardless of having AF^[4]^. The components of these scoring systems, such as advanced age, presence of hypertension, presence of diabetes mellitus, low ejection fraction, and female gender, have been associated with poor outcomes, including recurrent ischemic events after cardiac surgery^[5]^.

In this study, we aimed to investigate the predictive value of different thromboembolic risk scores in AF for the development of POAF.

## METHODS

### Study Population

This study included patients who underwent isolated CABG at the Suleyman Demirel University, Education and Research Hospital, between June 2017 and March 2019. The study population was retrospectively and consecutively analyzed by using our database, which was collected as a part of routine clinical practice. The overall study population included 400 patients undergoing CABG. Exclusion criteria were hyperthyroidism, age < 18 years, prior cardiac surgery, class III or IV heart failure, previous AF, left atrial diameter > 55 mm, left ventricular ejection fraction < 0.25, sepsis, heart rate < 60 bpm, systolic blood pressure < 90 mm Hg, inflammatory disease, and being already on antiarrhythmic treatment. According to these criteria, 30 patients were excluded due to previous AF (n=10), heart rate < 60 bpm (n=15), and hyperthyroidism (n=5). Therefore, 370 patients were included in this substudy. Informed consent was obtained from each patient, and the study protocol conforms to the ethical guidelines of the 1975 Declaration of Helsinki as reflected in a priori approval by the institution's human research committee (Date: 28.05.2019, Decision no: 181). Similar operative techniques were used in all of the patients.

### Diagnosis of Thromboembolic Risk

The CHA_2_DS_2_-VASc risk score is calculated by assigning a score of 1 point for each of the following conditions: congestive heart failure (ejection fraction < 40%), hypertension, age between 65 and 74 years, diabetes mellitus, vascular disease (myocardial infarction or peripheral arterial disease), and female gender; and a score of 2 points for the following conditions: history of stroke or transient ischemic attack (TIA) and age > 75 years. The score is then used to predict the risk of thromboembolism in non-valvular AF patients^[2]^. The maximum value of the score is 9.

The ATRIA score was developed from the ATRIA study cohort and calculated using the following: anemia (hemoglobin < 13 g/dL in men and < 12 g/dL in women) (3 points), severe renal disease (estimated glomerular filtration rate < 30 mL/min/1.73 m^2^) (3 points), age ≥ 75 years (2 points), prior bleeding, and hypertension. An ATRIA score of 0 to 3 is defined as “low risk”, a score of 4 is defined as “intermediate risk”, and a score ≥ 5 is defined as “high risk”^[3]^. The maximum value of the score is 12.

### Rhythm Follow-up

The rhythms were followed by continuous electrocardiogram monitoring during intensive care unit stay and by all-day Holter during the rest of hospitalization. A 12-lead electrocardiogram was recorded every morning routinely and whenever the patients had symptoms suggestive of dysrhythmia. AF was defined as an irregular rhythm with the absence of discrete *P* waves in the 12-lead electrocardiogram. An AF episode lasting five minutes during hospitalization was defined as POAF^[6]^. In case of POAF, antiarrhythmics and electrical cardioversion were allowed based on the discretion of the physician.

### Statistical Analysis

Statistical Package for the Social Sciences version 16.0 software package was used for statistical analyses in this study. Categorical variables were expressed as frequency (%) and compared using the χ^2^ test. Kolmogorov-Smirnov test was used to test the distribution of numeric variables; those with normal distribution were expressed as mean ± standard deviation and were compared with Student’s *t*-test. Data without normal distribution were expressed as median (interquartile range of 25%-75% percentiles) and were compared with the Mann-Whitney U test. In all statistical analyses, *P*-value < 0.05 was considered as statistically significant. The correlations between CHA_2_DS_2_-VASc and ATRIA risk scores, POAF, and other clinical, laboratory, and echocardiographic parameters were performed with Pearson’s or Spearman’s correlation analysis where appropriate. Univariate analysis and backward conditional binary logistic regression were performed to estimate the odds ratio (OR) and 95% confidence interval (CI) for the prediction of POAF. We carried out multivariate analysis in two models. Firstly, risk factors involved in CHA_2_DS_2_-VASc score were excluded from this analysis to avoid multicollinearity. Secondly, risk factors and other factors except CHA_2_DS_2_-VASc score were put to multivariate analysis. Receiver operating characteristics (ROC) curve analysis was used to analyze the prognostic value of CHA_2_DS_2_-VASc and ATRIA risk scores for POAF. C-statistic (area under the curve [AUC)] was presented as a unified estimate of sensitivity and specificity according to the cutoff value that was obtained by a ROC curve analysis. The optimal cutoff value was defined as the value yielding the maximal Youden index^[7]^, or the best combined sensitivity and specificity. All ROC comparisons were performed using the DeLong test^[8]^. C-statistic (AUC) was presented as a unified estimate of sensitivity and specificity.

## RESULTS

A total of 370 patients (mean age: 62.34±12 years; range, 28-84 years) were included in this study. During the follow-up period, 110 patients (29.7%) developed POAF. Demographic and clinical characteristics of the patients with and without POAF are listed in Table 1. Patients with POAF were significantly older and of the male gender when compared to patients without POAF (*P*<0.001 and *P*=0.03, respectively). Diabetes mellitus, hypertension, congestive heart failure, peripheral vascular disease, and stroke/transient ischemic event rates were higher in patients with POAF than in patients without POAF. There were no statistically significant differences between patients with and without POAF with regards to cholesterol parameters (for all parameters *P*>0.05). Left ventricular ejection fraction was significantly lower in patients with POAF than in patients without POAF (*P*<0.001). Preoperative fasting glucose levels were higher in patients with POAF than in patients without POAF (*P*<0.001).

**Table 1:** Demographic and clinical characteristics of patients with and without atrial fibrillation (AF).

	Without AF (n = 260)	With AF (n = 110)	*P*-value
Age (years)	59.8±12	68.1±8.4	< 0.001
Body mass index	29.0±5.3	28.0±4.5	0.114
Female gender (n, %)	82 (31.5)	24 (21.8)	0.03
Diabetes mellitus (n, %)	108 (41.5)	62 (56.4)	0.006
Hypertension (n, %)	184 (70.8)	100 (90.8)	< 0.001
Congestive heart failure (n, %)	12 (4.7)	20. (18.9)	< 0.001
Peripheral vascular disease (n, %)	70(26.9)	46(41.8)	0.004
History of CVA (n, %)	36 (13.8)	24 (21.8)	0.04
Ejection fraction (%)	56.5±9.4	49.2±11.6	< 0.001
Left atrial diameter (mm)	39.1±6.1	38.9±7.4	0.816
LVEDD (mm)	47.2±5.4	46.6±5.0	0.296
LVESD (mm)	29.6±6.8	31.3±5.7	0.026
IVSD (mm)	12.0±3.2	12.5±2.8	0.175
PWD (mm)	11.1±2.2	11.2±1.0	0.835
Aortic diameter (mm)	26.8±3.6	26.6±3.1	0.580
Total cholesterol (mg/dl)	201.2±41.3	196.9±39.4	0.412
HDL cholesterol (mg/dl)	41±10	42±9.8	0.713
LDL cholesterol (mg/dl)	126±40	125±34	0.915
Triglycerides (mg/dl)	160±106	142±49	0.209
Creatinine (mg/dl)	1.0±0.3	1.1±0.4	0.161
ALT (U/L)	22.2±12.2	27.5±29.8	0.015
AST (U/L)	31.1±23.6	33.0±22.7	0.489
Glucose (mg/dl)	142.5±67	198.6±119.9	< 0.001
CHA_2_DS_2_VASc score	2.2±1.4	3.1±1.9	< 0.001
ATRIA risk score	1.9±2.4	3.4±2.5	< 0.001

Data presented as mean ± standard deviation or number (%) of the patients. CHA_2_DS_2_-VASc score stands for congestive heart failure, hypertension, age ≥ 75 years, diabetes mellitus, previous stroke, vascular disease, age 65 to 74 years, and female gender. ALT=alanine aminotransferase; AST=aspartate aminotransferase; ATRIA=Anticoagulation and Risk Factors in Atrial Fibrillation; CVA=cerebrovascular accident; HDL=high-density lipoprotein; IVSD=interventricular septum diameter; LDL=low-density lipoprotein; LVEDD=left ventricular end-diastolic diameter; LVESD=left ventricular end-systolic diameter; PWD=posterior wall diameter

The postoperative drainage amounts in the first 24 hours and 48 hours were higher in patients with POAF than in patients without POAF (*P*=0.001 and *P*=0.05, respectively) (Table 2). Cardiopulmonary bypass time was longer in patients with POAF than in patients without POAF (*P*=0.02) but there is no statistically difference between patients with and without POAF with regards to clamp time. The duration of the hospitalization at the intensive care unit was longer in patients with POAF than in patients without POAF (*P*<0.001). There was no statistically difference between patients with and without POAF with regards to reoperation due to hemorrhage and intraoperative and in-hospital mortalities (for all parameters *P*>0.05) (Table 2).

**Table 2:** Operative and postoperative parameters of the groups.

	Without AF (n = 260)	With AF (n = 110)	*P*-value
Cardiopulmonary bypass time (min)	78.8±27.3	86.8±39.6	0.02
Clamp time (min)	47.6±15.4	47.0±16.4	0.766
24-hour drainage (ml)	276.5±137	344.5±239	0.001
48-hour drainage (ml)	145.3±82	162.7±71.1	0.05
Duration of the hospitalization at the intensive care unit (days)	2.1±0.4	2.6±1.4	< 0.001
Bypass number (n)	2.4±2	2.4±0.7	0.806
Reoperation due to hemorrhage (n, %)	4 (1.5)	2 (1.8)	0.575
Intraoperative mortality (n, %)	-	-	
In-hospital mortality (n, %)	8 (3.1)	4 (3.6)	0.500

AF=atrial fibrillation

The mean CHA_2_DS_2_-VASc and ATRIA scores were significantly higher in patients with POAF than in patients without POAF (3.1±1.9 *vs*. 2.2±1.4, *P*<0.001; 3.4±2.5 *vs*. 1.9±2.4, *P*<0.001; respectively).

### Prediction of Postoperative Atrial Fibrillation

**Fig. 1:**
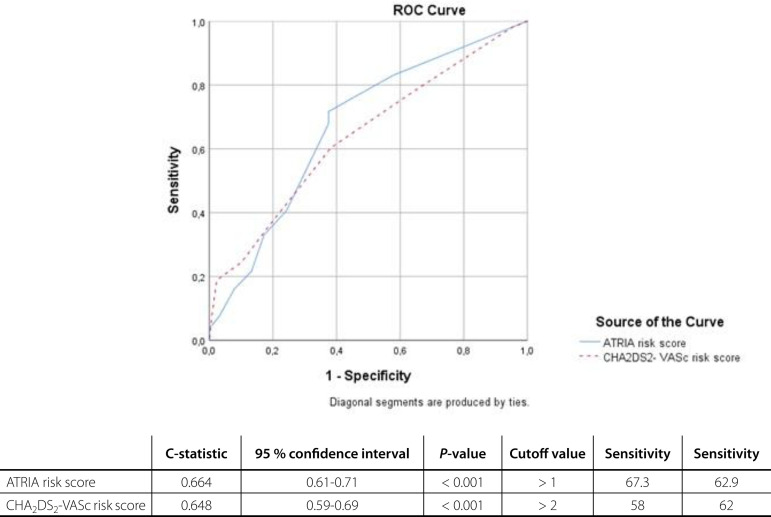
Receiver operating characteristics (ROC) curve analysis of risk scores. CHA2DS2-VASc stands for congestive heart failure, hypertension, age ≥ 75 years, diabetes mellitus, previous stroke, vascular disease, age 65 to 74 years, and female gender. ATRIA=Anticoagulation and Risk Factors in Atrial Fibrillation

Univariate analyses showed that high CHA_2_DS_2_-VASc and ATRIA risk scores, diabetes mellitus, hypertension, congestive heart failure, peripheral vascular disease, stroke/TIA, low left ventricular ejection fraction, advanced age, fasting glucose level, serum alanine aminotransferase (ALT) level, 24-hour drainage amount, pumping time, and male gender were significantly associated with a higher risk of development of POAF (Table 3). Firstly, multivariate binary logistic regression analysis including characteristics - except for CHA_2_DS_2_-VASc and ATRIA scoring systems - associated with new-onset AF in univariate analysis showed that fasting glucose level (OR 1.009; 95% CI 1.006-1.013; *P*<0.001), age (OR 1.10; 95% CI 1.06-1.14; *P*<0.001), left ventricular ejection fraction (OR 0.91; 95% CI 0.89-0.94; *P*<0.001), male gender (OR 1.94; 95% CI 0.93-4.07; *P*=0.076), 24-hour drainage amount (OR 1.002; 95% CI 1.001-1.004; *P*=0.002), and serum ALT level (OR 1.022; 95% CI 1.001-1.043; *P*=0.038) remained independent factors for incident AF (Table 4). After that, multivariate binary logistic regression analysis including characteristics - except for diabetes mellitus, stroke/transient ischemic disease, peripheral vascular disease, male gender, congestive heart failure, hypertension, age, and left ventricular ejection fraction - associated with new-onset AF in univariate analysis showed that ATRIA risk score (OR 1.232; 95% CI 1.11-1.36; *P*<0.001), fasting glucose level (OR 1.006; 95% CI 1.004-1.009; *P*<0.001), and 24-hour drainage amount were associated with new-onset AF following CABG (OR 1.002; 95% CI 1.001-1.004; *P*<0.001) (Table 4). ROC curve analysis showed that both ATRIA score (C-statistic 0.66; 95% CI 0.61-0.71; *P*<0.001) and CHA_2_DS_2_-VASc score (C-statistic 0.64; 95% CI 0.59-0.69; *P*<0.001) were significant predictors of POAF (Figure 1). We calculated that a cutoff point of 1 for ATRIA and 2 for CHA_2_DS_2_-VASc scores could estimate the presence of POAF with a sensitivity of 67% and 62% and a specificity of 58% and 62%, respectively. We performed a pairwise comparison of ROC curves and recorded that the predictive value of ATRIA risk score with regards to POAF development was similar to that of CHA_2_DS_2_-VASc score (by DeLong method, AUC_ATRIA_*vs*. AUC_CHA2DS2-VASc_ *z*-test=0.568, *P*=0.569)

**Table 3:** Univariate regression analysis of study variables.

	Odds ratio	Confidence interval	*P*-value
CHA_2_DS_2_-VASc risk score	1.38	1.21-1.59	< 0.001
ATRIA risk score	1.24	1.14-1.36	< 0.001
Fasting glucose level	1.007	1.004-1.009	< 0.001
Age	1.08	1.05-1.11	< 0.001
Male gender	1.65	0.97-2.78	0.06
ALT level	1.014	1.00-1.02	0.03
24-hour drainage	1.002	1.001-1.003	0.001
48-hour drainage	1.003	1.000-1.005	0.056
Left ventricular ejection fraction	0.93	0.91-0.96	< 0.001
Cardiopulmonary bypass time	1.008	1.001-1.015	0.03

CHA_2_DS_2_-VASc stands for congestive heart failure, hypertension, age ≥ 75 years, diabetes mellitus, previous stroke, vascular disease, age 65 to 74 years, and female gender. ALT=alanine aminotransferase; ATRIA=Anticoagulation and Risk Factors in Atrial Fibrillation

**Table 4:** Multivariate regression analysis of study variables.

	Model 1			Model 2		
	Odds ratio	Confidence interval	*P*-value	Odds ratio	Confidence interval	*P*-value
24-hour drainage	1.002	1.001-1.004	0.002	1.002	1.001-1.004	< 0.001
Age	1.10	1.06-1.14	< 0.001			
Male gender	1.94	0.93-4.07	0.076			
Left ventricular ejection fraction	0.91	0.89-0.94	< 0.001			
ATRIA risk score				1.232	1.11-1.36	< 0.001
Fasting glucose level	1.009	1.006-1.013	< 0.001	1.006	1.004-1.009	< 0.001
Serum ALT level	1.022	1.001-1.043	0.038			

Model 1: risk factors and other factors except CHA_2_DS_2_-VASc and ATRIA risk scores. Model 2: variables except risk factors involved in CHA_2_DS_2_-VASc and ATRIA risk scores. CHA_2_DS_2_-VASc stands for congestive heart failure, hypertension, age ≥ 75 years, diabetes mellitus, previous stroke, vascular disease, age 65 to 74 years, and female gender ALT=alanine aminotransferase; ATRIA=Anticoagulation and Risk Factors in Atrial Fibrillation

## DISCUSSION

The current study showed that higher CHA_2_DS_2_-VASc and ATRIA scores were independently associated with the development of AF in patients undergoing CABG; consequently, both scores could be helpful and appropriate scoring systems for predicting AF after CABG.

Previous studies showed that hypertension, diabetes mellitus, obesity, valvular disease, increased age, and left atrial characteristics, such as size, volume, and scarring, contribute to the development of POAF^[9]^. Similarly, hypertension and diabetes mellitus rates were higher in patients with POAF than without POAF. The risk factors of POAF are similar to the components of CHA_2_DS_2_-VASc and ATRIA risk scores^[1]^. Furthermore, these scores can be used to predict the risk of POAF. Kashani et al.^[10]^ showed that the CHA_2_DS_2_-VASc score can be used as a simple and useful tool for predicting POAF in patients with undergoing CABG. Borde et al.^[11]^ carried out a study including 729 CABG patients and showed that higher CHA_2_DS_2_-VASc scores predict POAF. Parallel results were attained by Chua et al.^[12]^, based on a prospective study including 277 patients undergoing CABG and/or valve procedures. Moreover, we showed that CHA_2_DS_2_-VASC score have been predicting AF following ST-elevation myocardial infarction and associated with epicardial fat tissue and mitral annular calcification^[13-15]^. The data of the current study corroborate with the results of previous studies. Additionally, we showed that the ATRIA score can also be used for the prediction of AF following CABG. Moreover, ATRIA score was similar with CHA_2_DS_2_-VASc score in predicting POAF.

POAF is a serious complication of CABG and is associated with worse clinical outcomes such as prolonged length of hospital stay, rising costs, and increased short- and long-term morbidity and mortality^[16]^. Several guidelines recommended suggestions for prophylaxis against POAF^[17-19]^. However, these suggestions reveal unnecessary exposure to medications and for this reason, many physicians do not regularly apply them. These propositions may expose up to 70% of undergoing CABG patients to antiarrhythmic drugs and their subsequent side effects^[20]^. In a randomized study, Ozaydın et al.^[6]^ showed that carvedilol plus N-acetylcysteine decreased POAF incidence compared with metoprolol or carvedilol. Therefore, it is important to anticipate which patients may develop POAF. In this study, we demonstrated that CHA_2_DS_2_-VASc and ATRIA scoring systems could be used to determine patients at the highest risk of developing POAF, thus these scoring systems may decrease nonselective prophylaxis.

Additionally, the present study showed that older age, male gender, drainage amount, pumping time, and ALT levels were associated with POAF. The results corroborated with the previous studies^[1,6,9,16,19,21]^. Age is the most common risk factor of POAF because of occurred myocardial fiber loss, fibrosis, and collagen deposition^[22]^. Previous studies showed that cross-clamp duration and pumping time are associated with POAF, and reducing cross-clamp duration may reduce POAF^[1,21,23]^.

### Limitations

This study has several limitations: it has a single-center design, it is nonrandomized study, and the sample size was small. Additionally, we recruited patients with AF for at least five minutes. In previous studies, patients with AF may be separated according to AF duration.

## CONCLUSION

In conclusion, we have shown in the current study that ATRIA and CHA_2_DS_2_-VASc scoring systems were useful for detecting AF following CABG. Additionally, when the ATRIA risk score was compared with the CHA_2_DS_2_-VASc scoring system, it was found to be powerful in predicting the development of POAF. Patients at high risk according to ATRIA and CHA_2_DS_2_-VASc scoring systems should be followed up for rhythm disturbance after the procedure and it should be administrated the suggestions of the guidelines prior to the procedure.

**Table:** 

Authors' roles & responsibilities	
DU FA EI	Substantial contributions to the conception or design of the work; or the acquisition, analysis, or interpretation of data for the work; final approval of the version to be published Substantial contributions to the conception or design of the work; or the acquisition, analysis, or interpretation of data for the work; final approval of the version to be published Substantial contributions to the conception or design of the work; or the acquisition, final approval of the version to be published
